# Novel Fibrinolytic Protease Producing *Streptomyces radiopugnans* VITSD8 from Marine Sponges

**DOI:** 10.3390/md17030164

**Published:** 2019-03-13

**Authors:** Dhamodharan D, Jemimah Naine S, Merlyn Keziah S, Subathra Devi C

**Affiliations:** 1 Department of Biotechnology, School of Bio Sciences and Technology, Vellore Institute of Technology, Vellore-632014, Tamil Nadu, India; dhamodharan@live.co.uk (D.D.); jemi.micro@gmail.com (J.N.S.); merlynkezi@gmail.com (M.K.S.); 2 Department of Life Sciences, Kristu Jayanti College, Bengaluru-560043, Karnataka, India

**Keywords:** marine sponges, marine actinomycetes, *Streptomyces radiopugnans* VITSD8, fibrinolytic protease, clot busters

## Abstract

Fibrinolytic enzymes have received more attention due to their medicinal potential for thrombolytic diseases. The aim of this study is to characterize the in vitro fibrinolytic nature of purified protease producing *Streptomyces radiopugnans* VITSD8 from marine brown tube sponges *Agelas conifera*. Three varieties of sponge were collected from the Rameshwaram Sea coast, Tamil Nadu, India. The fibrinolytic activity of *Streptomyces* sp. was screened and determined by casein plasminogen plate and fibrin plate methods respectively. The crude caseinolytic protease was purified using ammonium sulfate fractionation, affinity and ion-exchange chromatography. Based on the morphological, biochemical, and molecular characterization, the isolate VITSD8 was confirmed as *Streptomyces radiopugnans*. Maltose and peptone were found to be the best carbon and nitrogen sources for the production of fibrinolytic protease. The carbon and nitrogen source peptone showed (781 U/mL) enzyme activity. The optimum pH and temperature for fibrinolytic protease production was found to be 7.0 and 33 °C respectively. The purified enzyme showed a maximum specific activity of 3891 U. The blood clot lysis activity was compared with the standard, and it was concluded that a minimum of 0.18 U (10 µL) of purified protease was required to dissolve the blood clot. This is the first report which exploits the fibrinolytic protease activity of *Streptomyces radiopugnans* VITSD8 extracted from a marine sponge. Hence the investigation suggests a potential benefit of purified fibrinolytic protease which will serve as an excellent clot buster alternative.

## 1. Introduction

As per a WHO report, about 17 million individuals are supposed to die due to cardiovascular infections. The frequency of the intense myocardial localized necrosis is increasing around the world [1]. Thrombus develops inside arteries, leading to thrombosis, heart stroke, and pulmonary embolism [2]. Therefore, thrombolytic treatments have become a traditional remedy regarding acute myocardial infarction (AMI). Synthetic therapeutics used in such disorders has serious adverse effects, so there is a need to investigate new and safe natural thrombolytic agents [3]. Protein treatments are turning out to be more pervasive in present day solutions for AMI. Since a wide source is available, microbial fibrinolytic enzymes have attracted a great deal of therapeutic enthusiasm during the last decade [4]. For every fibrinolytic protease, there is a unique spotlight on the protein structure and activity. Proteases guarantee no side-effects to patients with inborn shortcomings. Hence, drug screening programs focused on microbial enzymes are found to be better alternatives. The discoveries of new members of actinomycetes and novel metabolites from marine environments have drawn attention towards marine sponges and sediments [5]. It has been reported that marine actinomycetes are extensive sources of metabolites with anti-viral, anti-bacterial, anti-tumor, anti-helminthic, insecticidal, immune-modulatory, and immunosuppressant properties when compared to terrestrial actinomycetes [6]. Marine actinomycetes were mostly isolated from sand, water, rock, and mangrove sediments. The colonies have aerial mycelium powdery appearance distinguished from bacteria; they are gram positive and filamentous in nature [7]. Actinokinase (AK) enzyme isolated from *Streptomyces megasporous* SD5 has shown thrombolytic activity similar to that of urokinase from human urine, which is fibrin specific. This extracellular protein is composed of 242 amino acids with a molecular weight of 35 kDa [8]. Fibrinolytic protease producing *Streptomyces violaceus* VITYGM from marine soil samples [9] and *Streptomyces venezuela* from marine water samples [10] were also reported previously. The expanding capability of AK applications elevated us to screen for novel and AK delivering from marine creatures. This offers extensive points of interest over the other reported marine *Streptomyces* sp. in view to the significance of the marine system which provides a rich source of novel actinomycetes and the increased industrial applications of the enzymes. Hence the present study aims to study the novel fibrinolytic enzyme producing actinomycetes isolated from marine sponges. To the best of our knowledge, this is the first report given on *Streptomyces* sp. from marine brown tube sponge *Agelas conifera* for a new potential fibrinolytic actinokinase.

## 2. Materials and Methods

### 2.1. Chemicals

Bovine serum albumin, tyrosine, thrombin, and standard streptokinase were obtained from Sigma Aldrich (Bengaluru, India). Starch casein agar, ISP broth, glucose, yeast extract, ammonium sulfate, manganese chloride, sodium carbonate, sodium bicarbonate, magnesium sulfate, sodium acetate, sodium chloride, sodium hydroxide, and ferrous sulfate were purchased from Hi Media laboratories (Mumbai, India). Copper sulfate, Folin–Ciocalteau reagent, potassium dihydrogen phosphate, trichloroacetic acid (TCA), hydrochloric acid (HCl), ethylene-diaminetetraacetic acid (EDTA), and Tris-HCl were purchased from Sisco research laboratories (SRL, Mumbai, India).

### 2.2. Sample Collection and Isolation of Actinomycetes

#### 2.2.1. Collection of Sponges

Three varieties of sponges were collected from the Rameshwaram Sea coast positioned on the eastern coast line, 28 km from Ramanathapuram with 9°281′ latitude and 79°301′ longitude. Sponges were collected in sterile polythene bags. The sponges were distinguished by their morphological appearance [11]. Following collection, many epiphytic faunas were removed with sterile marine water in order to remove microbes originating from ecological marine normal water.

#### 2.2.2. Spicule Preparation

For spicule preparation, the sponge fragments were taken in a test tube and 10 volumes of fuming nitric acid (HNO_3_) were added and kept for steaming, till the cell product was smeared [12,13].

#### 2.2.3. Isolation and Screening for *Streptomyces* sp. from Marine Sponges

Sponges were portioned into smaller items by using sterile and clean scissors. They were smashed with a sterile mortar and pestle. Serially diluted around 10^−6^ and inoculated in Starch casein agar plate. The fibrinolytic activity of *Streptomyces* sp. was screened by casein plate assay.

### 2.3. Bacterial Strain and Culture Conditions

*Streptomyces* sp. was cultured in a medium containing (g/L): starch, 10; K_2_HPO_4_, 2; KNO_3_, 2; NaCl, 2; Casein, 0.3, and remaining trace amount salts were MgSO_4_, CaCO_3_, and FeSO_4_. Cultivation was carried out for 48 h on a rotatory shaker set at 33 °C and 140 rpm [14].

### 2.4. Determination of Protease Activity by Caseinolytic Method

Protease activities of the isolates were determined by standard caseinolytic assay [15].

### 2.5. Quantification of Protein and Enzyme Activity

Protein content was determined by Lowry’s method [16] using bovine serum albumin as the standard protein. Protease activity was determined by Folin–Ciocalteu method using tyrosine as the standard [17].

### 2.6. Characterization of Selected Streptomyces sp.

The selected strains were characterized according to the method employed by collaborators in the International *Streptomyces* Project [18].

#### 2.6.1. Molecular and Phylogenetic Analysis

Total genomic DNA was isolated using the phenol chloroform method [19]. PCR amplification of 16S rDNA was done. The PCR product was detected by agarose gel electrophoresis. The sequence was subjected to a homology search using the BLAST program of the National Center for Biotechnology Information (NCBI). The acquired sequences were used for a gene homology search, with the 16S rDNA sequences available in the public databases from BLAST and identified to the generic level. The 16S rDNA sequences aligned from the Gen Bank database and a phylogenetic tree was constructed via the EvolView program [20].

#### 2.6.2. Scanning Electron Microscope (SEM) Analysis

*Streptomyces* sp. from the plate was streaked onto inclined coverslips [21]. These coverslips were subjected to a vacuum evaporator provided with a variable tilt, rotary specimen support. The metal-plated coverslips were examined with a Scanning Electron microscope Model “Zeiss EVO 18”, Oberkochen, Germany.

### 2.7. Optimization

A total of four different carbon sources, maltose, dextrose, oat meal, and starch, were used, the nitrogen sources casein, yeast extract, peptone, and L-aspargine were used for optimization. The optimum temperature and pH were 33 °C and 7.2 respectively. The temperature and pH were kept constant for carbon-nitrogen source optimization. The effects of pH and temperature were determined by the Folin–Ciocalteu method. The effect of pH varied from (3–11) using standard buffers: citric acid–sodium phosphate (pH 3–7), Tris–HCl (pH 7.5–9), and NaHCO_3_ (pH 9.6–11). To determine the pH stability of the enzyme, aliquots of enzyme samples were incubated with the respective buffers for 3 h at 18 °C, and the residual activities were measured at pH 7.2. The temperature optimum of *Streptomyces radiopugnans* VITSD8 was assessed by carrying out the enzyme assay at various temperatures (4–60 °C) at pH 7.2.

### 2.8. Purification

*Streptomyces radiopugnans* VITSD8 was cultured for 48 h, centrifuged at 10,000 rpm for 1 h and solid (NH_4_)_2_SO_4_ at 0–85% saturation was added to the supernatant to precipitate the proteins. Proteins were recovered by centrifugation at 8000 rpm for 15 min at 4 °C, dialyzed against 10 mM Tris–HCl buffer (pH 7.2) and concentrated with an ultra-filtration membrane (Millex syringe filter Darmstadt, Germany). The enzyme was then subjected to gel filtration on a Sepharose CL-6B column (120 cm 2.2 cm), pre-equilibrated with 10 mM Tris–HCl buffer (pH 7.2) at a flow rate of 15 mL/h. The active fractions with high specific activities were pooled, concentrated, and further purified using a Poros-HQ ion exchange column (10 cm × 1 cm), pre-equilibrated with 10 mM Tris–HCl buffer (pH 7.2). The bound proteins were eluted with a gradient of 10 mM Tris–HCl buffer (pH 7.2) containing 0–1 M HCl at a flow rate of 30 mL/h [22]. The molecular weight of the obtained enzyme was determined by sodium dodecyl sulphate-polyacrylamide gel electrophoresis (SDS-PAGE) [23]. The protein standard albumin (66.2 kDa), ovalbumin (45 kDa), and carbonic anhydrase (29 kDa) were used as markers.

### 2.9. Blood Clot Lysis Assay

Clot lysis activity of the enzyme was determined by modified Holmstrom method [24].

### 2.10. Fibrin Plate Method

The fibrin plate method was used to determine the activity of purified *Streptomyces* protease against the clot [25].

### 2.11. Partial Clot Lysis Assay

Clot lysis activity of the purified enzyme was determined by the percentage of red blood cells (RBCs) released at 275 nm. The percentage of total clot lysis was calculated with reference to the calibrated curve of RBCs used in the clot, in which the 100% was equivalent to all the RBCs in the clot being released into the surrounding fluid [2].

### 2.12. HPLC Analysis

The fractions obtained were analyzed for protein purity by HPLC using a C18 column, UV Detector-2487 (Waters HPLCPump-1525, Milford, MA, USA). The mobile phase acetonitrile and water were in the ratio of 1:1, and purity was validated at 280 nm.

### 2.13. FTIR Analysis

FTIR spectra of samples were measured using FTIR spectrometer (IR Affinity-1Shimadzu, Kyoto, Japan). Approximately 0.5 mg of the sample was mixed with 1% of potassium bromide and compressed to get a pellet and analyzed [26].

## 3. Results and Discussion

**Figure 1:**
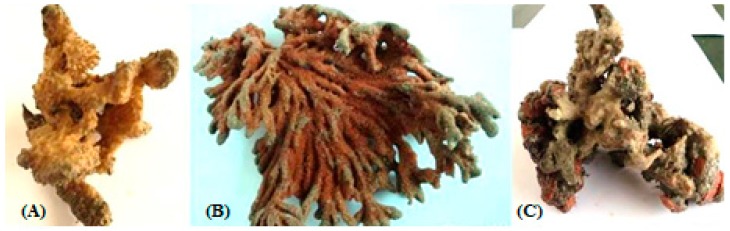
Three different kinds of sponges taken from the Rameshwaram coastal area, Tamil Nadu, India, (**A**) *Niphates erecta*, (**B**) *Desmapsamma anchorata* and (**C**) *Agelas conifer*.

**Figure 2:**
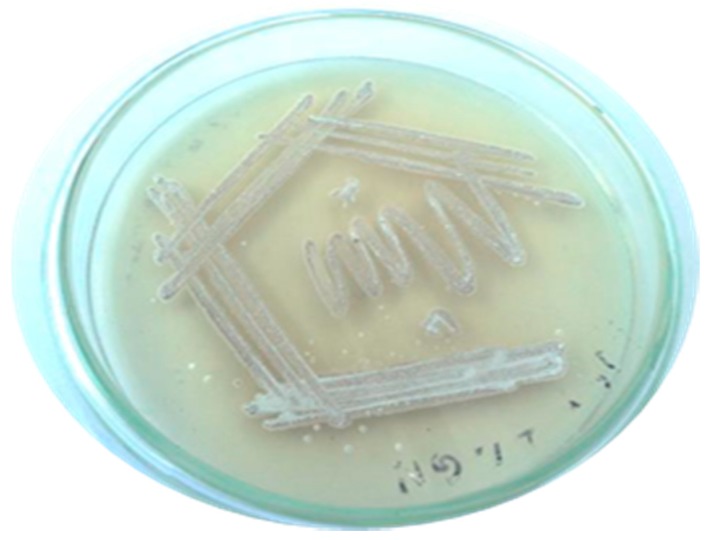
Pure culture of *Streptomyces radiopugnans* VITSD8.

**Figure 3:**
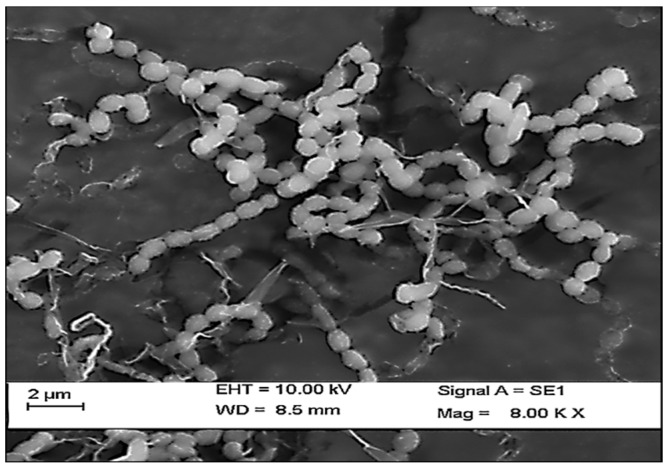
Scanning electron micrograph representing the spore chain morphology of *Streptomyces radiopugnans* VITSD8.

In the present study, a total of three species of sponge were collected from Rameshwaram, Tamil Nadu, India. The species of the sponges were identified by their characteristic morphological appearance and structural spicule appearance on observing spicules prepared with 10% formalin. All the three marine sponges were identified, as *Niphates erecta* (Niphates), *Desmapsamma anchorata* (Desmacididae), and *Agelas conifera* (Agelasidae) [27]. *Niphates erecta* (Niphates), commonly known as lavender rope sponge, has a characteristic appearance of greyish brown color with irregular distribution of oscules (Figure 1A), *Desmapsamma anchorata* is soft and slimy sponge lumpy growing sponge with volcano-shaped oscules on the elevations (Desmacididae) (Figure 1B), and *Agelas confiera* (Agelasidae) is a brown tube sponge with a characteristic stiff consistency, aerial hyphae morphology grey color (Figure 1C) [11]. They are classified according to the specific spicule of each species and they have monoaxonic and tetra axonic silicious spicules [27]. They are structurally colored. The strain VITSD8 was isolated from the sponge *Agelas confiera*, and had monoaxonic and tetra axonic silicious spicules. The isolation of *Streptomyces* sp. was carried out using starch casein agar. A total of 68 colonies were isolated from three species of marine sponges. Only 16 strains were selected based on their mycelium color. The isolated colonies had a powdery appearance with a characteristic feature of a warty surface and spiral chains when under a scanning electron microscopy (magnification, 6000) with a spore diameter of 0.6 µm (Figure 2 and Figure 3; Table 1 and Table 2).

The molecular weight of actinokinase is 38 kDa and the low molecular weight enlightens the high efficiency of fibrin degradation. The enzyme is serine endopeptidase, classified as a hydrolase type of enzyme, which breaks the fibrin and dissolves the clot.

The strain was observed to produce large quantities of double shaped aerial mycelium on yeast extract malt extract agar (ISP 2) when compared to other ISP extract agar (Table 3).

**Figure 4:**
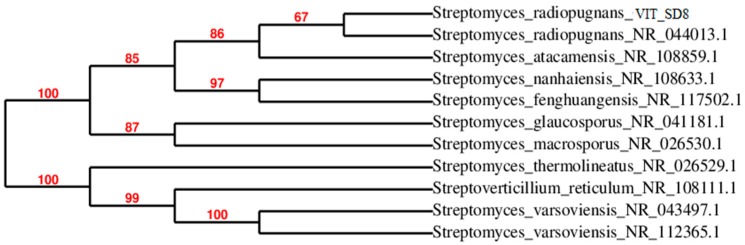
Phylogenetic tree of *Streptomyces radiopugnans* VITSD8.

Sequence results of 16S rDNA were exported to the database and checked for homologous alignment. Based on the alignment results, strain VITSD8 was found to be *Streptomyces radiopugnans* which showed 99% similarity. The strain name was designated as *Streptomyces radiopugnans* VITSD8. The partial 16S rDNA sequence was deposited in Gen Bank under the accession number KR233811.1. The phylogenetic tree was depicted based on 16S rDNA of the strain *Streptomyces radiopugnans* VITSD8 (Figure 4).

**Figure 5:**
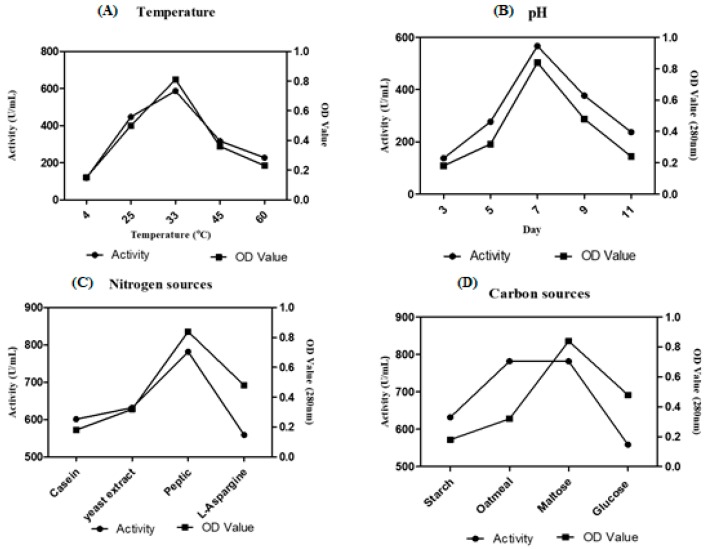
Optimization of (**A**) Temperature (**B**) pH (**C**) carbon sources (**D**) nitrogen sources.

Among eight *Streptomyces* sp. screened for caseinolytic activity, VITSD8 isolate showed promising results representing 12 mm of zone of hydrolysis. *Streptomyces radiopugnans* VITSD8 grown on medium supplemented medium with maltose as the carbon source and peptone as the nitrogen source showed a better activity of 781 U/mL. The strain grown in the medium at pH 7 and temperature 33 °C showed 567 U /mL and 587 U/mL activity (Figure 5).

**Figure 6:**
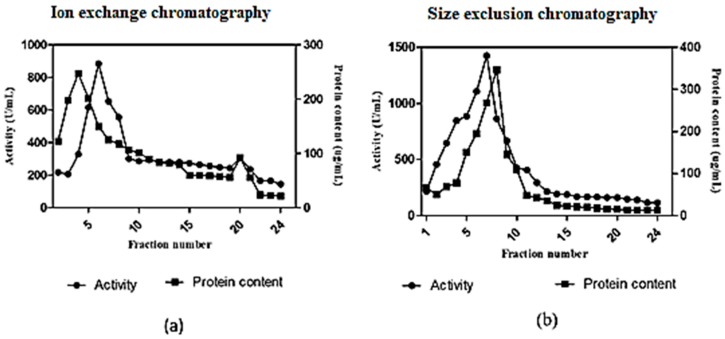
(**a**) Elution profile of *Streptomyces radiopugnans* VITSD8 for Ion exchange with Poros-HQ column (**b**) elution profile of *Streptomyces radiopugnans* VITSD8 for a size exclusion chromotography with Sepharose CL-6B column. The enzyme activity was expressed in terms of U/mL.

In anion exchange chromatography using DEAE cellulose, the fibrinolytic activity was eluted in 24 fractions (Figure 6a). The 5th elution sample showed a maximum activity of 884 U/mL. The active fraction was further purified by size exclusion chromatography using a Sephadex G100 column. The maximum fibrinolytic activity of the purified enzyme was found to be 1426 U/mL (Figure 6b). The purification fold of enzyme produced from *Streptomyces radiopugnans* VITSD8 is represented in Table 4. The fibrinolytic activity of protease from *S. radiopugnans* VITSD8 was increased by 35-fold after purification, and the final yield was 22.36%.

The enzyme was purified to homogeneity resulting in 22.36-fold purification and 35% activity recovery. From the purification table, the specific activity of marine *Streptomyces radiopugnans* VITSD8 is 3891 U/mg, which is higher than the specific activity of 2563.6 U/mg of terrestrial *Streptomyces* sp. P3 strain isolated from soil [28].

**Figure 7:**
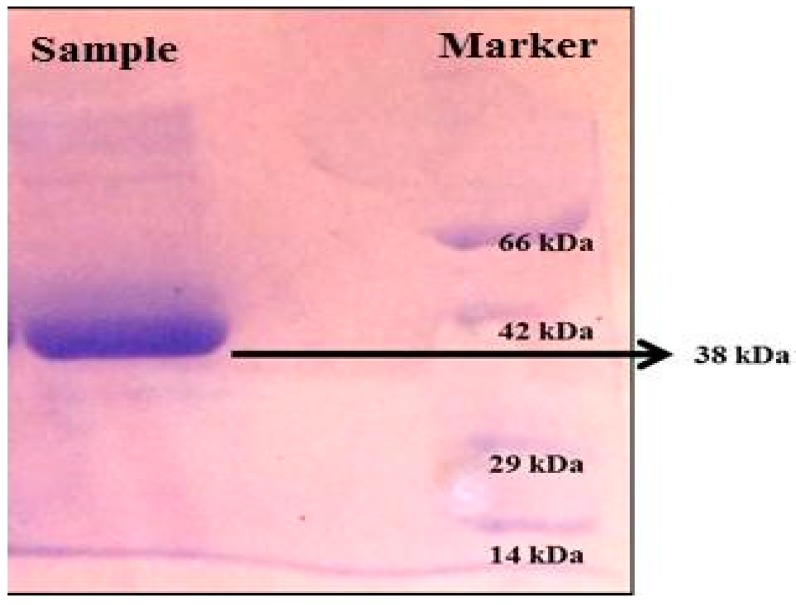
SDS-PAGE analysis of protein produced from *Streptomyces radiopugnans* VITSD8 (Sample-38 KDa protein).

SDS-PAGE analysis presented a single band in the sample corresponding to a molecular weight of 38 kDa (Figure 7).

**Figure 8:**
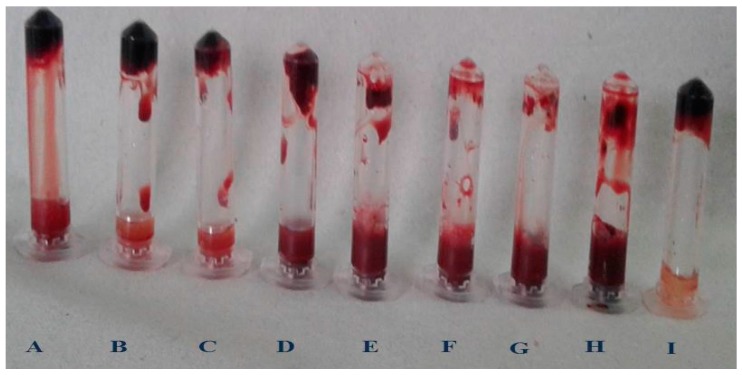
Blood clot lysis of Purified fibrinolytic protease from *Streptomyces radiopugnans* VITSD8 strain. (A—40%, B—50%, C—60%, D—70%, E—80%, F—90%, G—100%, H—PC (streptokinase), and I- NC (buffer).

**Figure 9:**
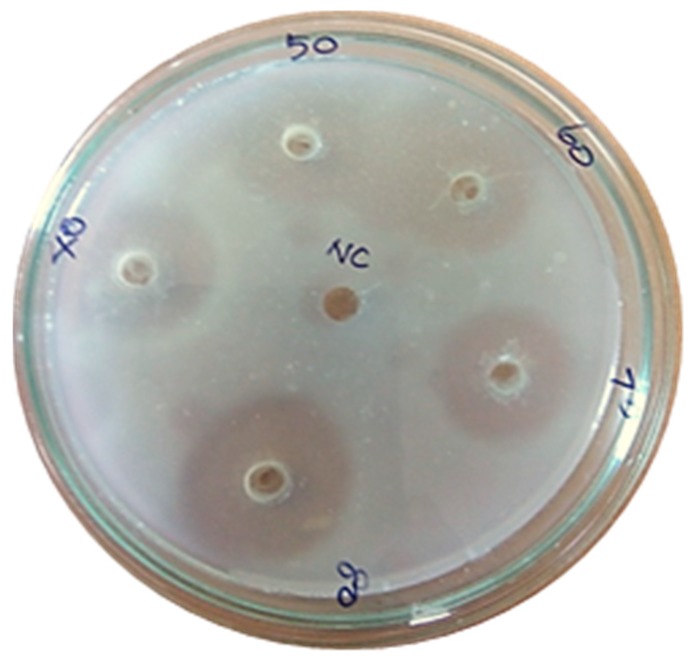
Casein plasminogen assay (80% of purified fibrinolytic protease showed 30 mm of clear zone).

An electrophoretic analysis of *Streptomyces radiopugnans* VITSD8 was carried out in 12% (*w*/*v*) polyacrylamide slab gel according to the method of Laemmli. Gels were stained with Coomassie Brilliant Blue R-250 and destained with ethanol/glacial acetic acid/distilled water (1:1:8, by vol.). Protein marker sizes in kDa are shown at the right of the lane and purified proteins from *Streptomyces radiopugnans* VITSD8 at left side. Based on the results it was found that the enzyme could lyse both natural clots as well as synthetic clots of fibrinogen, plasmin, and thrombin. A very high variation in clot lysis activity of purified enzyme and positive control (streptokinase) was observed (Figure 8). The fibrin plate method was used to determine the activity of purified fibrinolytic protease against the clot. The clear zone of hydrolysis on a fibrin degradation agar plate of 30 mm indicates the fibrinolytic activity of the enzyme (Figure 9). The release of RBCs was 100% with purified *Streptomyces radiopugnans* VITSD8 fibrinolytic protease at the 20th and 30th min of incubation, while the control clot indicated the release of RBCs at 30 min (Table 5).

**Figure 10:**
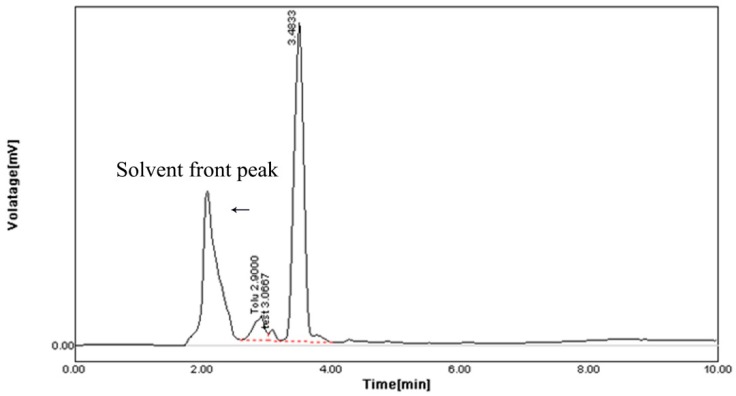
HPLC analysis of purified fibrinolytic protease from *Streptomyces radiopugnans* VITSD8 strain. The retention time is 3.48 min.

**Figure 11:**
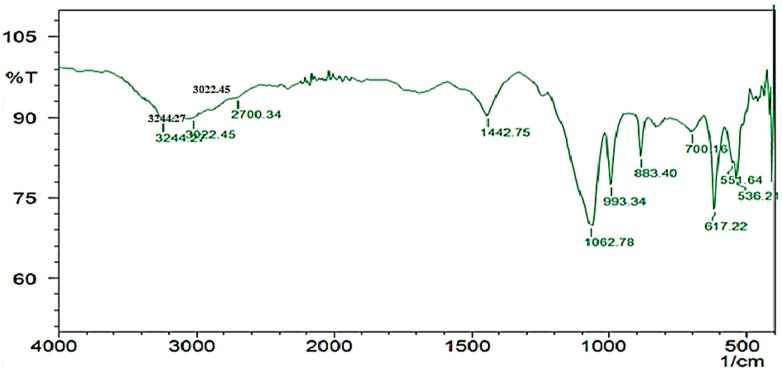
FTIR profile of purified fibrinolytic protease from *Streptomyces radiopugnans* VITSD8. The frequency of 1062.78 indicates the presence of a strong aliphatic group in the *Streptomyces radiopugnans* VITSD8 protease.

The HPLC profile represented the retention time at 3.48 min and it showed a single peak, indicating the purity of enzyme (Figure 10). The FTIR profile of the purified fibrinolytic protease showed a characteristic O-H stretch at 3244 cm^−1^, followed by carboxylic acid O-H stretch at 2700 cm^−1^. The peak at 1422 cm^−1^ was attributable to a carbonyl stretching vibrations. In addition, the peak at around 1062 cm^−1^ is attributed to C-C bond stretching. Furthermore, the peak at 993 cm^−1^ was attributed to an aromatic binding and the peak at 617cm^−1^ corresponded to C-Cl stretching (Figure 11).

Some of the previous studies reported in fibrinolytic protease from actinomycetes includes *Streptomyces spheroids* M8 [15] and *Actinomyces thermovulgaris* [17]. The cloning and expression of the thermophilic *Streptomyces* actinokinase gene has also been reported [29]. A purified enzyme from thermophilic *Streptomyces megasporus* SD5 showed a potential fibrinolytic property at 0.11 mg crude enzyme with a specific activity of 4.2 U [29]. Post-electrophoretic reactivity revealed a monomeric form of the enzyme with a molecular weight of 35 kDa. The optimum pH and temperature for production of the enzyme was 8 and 55 °C, respectively. The enzyme was resistant to a broad range of pH ranging from 6 to 9 and temperature ranging from 37 °C to 60 °C. Hence the study also justifies and confirms the characteristics of the fibrinolytic enzyme which mimics actinokinase; further conformational studies will help for a better understanding of its functional properties.

Marine microorganisms have been reported to have fibrinolytic, caseinolytic, and albuminolytic properties [30]. The fibrinolytic proteases, especially actinokinases, are mostly produced from non-pathogenic actinomycetes. It is a neutral enzyme. They have higher specificity towards N-succinyl-Ala-Ala-Pro-Phe-pNA, a substrate of chymotrypsin [31]. Actinobacteria, *Streptomyces rimosus*, has been reported for its maximum fibrinolytic activity of 800 U/mL [32]. A thermophilic actinoprotease produced by *Streptomyces* sp. isolated from soil samples of the Khartoum region of Sudan has been reported to have efficient fibrinolytic activity [30]. Similarly, reports on *Streptomyces rubiginosus* VITPSS1 [33] also stated that fibrinolytic enzymes from marine actinomycetes act faster than the commercially existing enzymes and instigate further to explore more fibrinolytic enzyme producing marine *Streptomyces* sp.

## 4. Conclusions

This is the first report on fibrinolytic protease producing *Streptomyces radiopugnans* VITSD8 isolated from marine sponges. The results of the study strongly suggest that the protein is a fibrinolytic enzyme. The enzyme degraded the clot by direct fibrinolysis more efficiently than the positive control (streptokinase). This fibrinolytic enzyme was highly active and stable in moderate pH and temperature. It can be further developed as a potential candidate for thrombolytic therapy in clinical trials. A clot-busting enzyme provides new hope for cancer, stroke, and heart patients. Enzymes from natural marine isolates are the important pharmacological substances which can be used for developing new and effective fibrinolytic agents. The outcome of the current research would present a new window of opportunity for the treatment of cardiovascular diseases. Furthermore, the identification and characterization of proteins would lead to the possibility of developing drugs, and thus making a better formulation, to be used for treatment. To our knowledge, this is one among the few studies on fibrinolytic investigation of enzymes in vitro. Hence studies on actinobacteria will lead to identifying and establishing the most fibrinolytic enzymes which will be effective against cardiovascular diseases. In the near future, the strain *Streptomyces radiopugnans* VITSD8 will serve as a potential source for the commercial production of fibrinolytic enzymes.

## Figures and Tables

**Table 1:** Biochemical and physiological properties of *Streptomyces* sp. VITSD8.

Microscopy		Corn Steep Liquor	+
Grams Stain	+	Sodium acetate	+
Colony Pigmentation	-	Sodium citrate	-
Melanin Pigmentation	-	Urea	-
Motility	-	Effect of Temperature	
Soluble Pigment	-	15 °C	-
Carbon Source	-	28 °C	+
D-glucose	+	37 °C	+
D-galactose	-	45 °C	-
Mannose	+	Effect of pH	
Maltose	+	5	-
Lactose	+	6	-
Trehalose	+	7	+
Melibose	-	8	-
Amino Acid Utilization		9	-
Cysteine	+	NaCl Concentration	
Arginine	+	1%	+
Therionine	+	2%	+
Alanine	+	3%	-
Aspartic acid	+	5%	-
Glycine	+	10%	-
Histidine	-	Hydrolysis	
Lysine	+	Starch Hydrolysis	+
Phenyl alanine	+	Nitrate Reduction	-
Tryptophan	+	Gelatin Liquefaction	+
Methionine	+	Hemolysis on Blood Agar	+
Isoleucine	-	Esculin Hydrolysis	-
Valine	-	H_2_S	-
Ornithine	-	Antibiotic Resistance	
Asparagine	-	Streptomycin	S
Nitrogen Source		Tetracyclin	S
Peptone	+	Bacitracin	R
Yeast Extract	+	Kanamycin	S
Casein	+	Ampicillin	R
Ammonium Sulphate	+	Gentamicin	R
Ammonium Nitrate	+	Rifampacin	R
Ammonium Citrate	+	Vancomycin	S
Beef Extract	+	Fluconazole	R

**Table 2:** Phenotypic properties separating the strain VITSD8 from related species according to the nonomura key.

Characteristic Features	*Streptomyces* sp. VITSD8	Utilization of Carbon Sources	
Spore surface	Smooth	Arabinose	₊
Spore chain morphology	Rectiflexibles	Xylose	₋
Aerial mycelium	Grey	Inositol	₊
Melanoid pigment	-	Mannitol	₊
Reverse side pigment	-	Fructose	₊
Soluble pigment	-	Rhamnose	₊
		Sucrose	₊
		Raffinose	₊

**Table 3:** Cultural characteristics of strain *Streptomyces* sp. VITSD8 on various media.

Medium	Growth	Aerial Mycelium	Substrate Mycelium
Tryptone-Yeast Extract Broth (ISP medium 1)	Good	White	Yellow
Yeast-malt extract (ISP medium 2)	Very good	White	Yellow
Oatmeal agar (ISP medium 3)	Good	White	Yellow
Inorganic salt starch agar (ISP medium 4)	Good	Grey	Grey
Glycerol-aspargine agar (ISP 5)	Good	White	Ash
Peptone yeast extract iron agar (ISP medium 6)	Slow	Grey	Grey
Tyrosine agar base (ISP medium 7)	Good	Ash	Ash
Nitrate agar (ISP 8)	Good	Ash	Ash
Carbon utilization agar (ISP medium 9)	Good	White	Grey
Bennett agar	Good	Grey	Grey
Nutrient agar	Slow	White	White
Czapex Dox agar	Good	White	White
*Kenknight’s agar*	Good	White	Grey
Actinomycete isolation agar	Very good	White	Grey
Starch casein agar	Very good	Grey	Grey

**Table 4:** Purification of fibrinolytic protease.

Fractions	Total Protein (mg)	Relative Activity (U)	Total Activity (U·mL)	Specific Activity (U·mg^−1^)	Fold Purification	% Yield
**Crude enzyme**	71	247	12,350	174	1	100
**Ammonium sulphate precipitation**	15	545	7085	473	2.7	57
**Dialysis**	8	697	6273	784	4.51	51
**Ion-exchange chromatography**	3	884	5304	1768	10.16	43
**Size exclusion chromatography**	1.1	1427	4281	3891	22.36	35

**Table 5:** Percentage of Red blood cells (RBCs) released in partial clot lysis assay.

Time	10 min (%)	20 min (%)	30 min (%)
**Purified**	91	100	100
**Partially Purified**	57	78	86
**Ammonium Precipitate**	18	42	73
**Streptokinase**	87	94	100
